# Analysis of Salivary Microbiome and Its Association With Periodontitis in Patients With Obstructive Sleep Apnea

**DOI:** 10.3389/fcimb.2021.752475

**Published:** 2021-12-07

**Authors:** Yanlong Chen, Xuehui Chen, Xin Huang, Ying Duan, He Gao, Xuemei Gao

**Affiliations:** ^1^ Department of Orthodontics, Peking University School and Hospital of Stomatology, Beijing, China; ^2^ Department of Sleep Medicine, Airforce Medical Center, Beijing, China

**Keywords:** obstructive sleep apnea, salivary microbiome, periodontitis, 16S rRNA gene sequencing, periodontal pathogens

## Abstract

**Objectives:**

This study aimed to analyze the periodontal conditions of patients with obstructive sleep apnea (OSA) in relation to the salivary microbiome.

**Materials and Methods:**

In total, 54 male adults (27 with OSA, 27 controls) completed this cross-sectional study. All participants were monitored by overnight polysomnography (PSG) and underwent full-mouth periodontal examination. Saliva samples were then collected, and the microbial 16S ribosomal RNA gene was sequenced. The data were analyzed to determine the microbial distribution and the community structure of the two groups.

**Results:**

Demonstrated by alpha and beta diversity, the OSA group had a lower microbial richness and a lower observed species than the controls. There was no significant difference in the microbial species diversity or evenness between the OSA and the non-OSA groups. The OSA group had fewer operational taxonomic units (OTUs), and the distribution of microbiome showed that several gram-positive bacteria had higher abundance in the OSA group. As for periodontal pathogens, the relative abundance of *Prevotella* was significantly increased in the OSA group. No significant difference was observed in the relative abundance of other pathogens at either the genus or species level.

**Conclusions:**

The salivary microbial community structure was altered in patients with OSA in terms of species richness and trans-habitat diversity, along with an increase in *Prevotella*, a specific periodontal pathogen. These findings might explain the high prevalence of periodontitis in OSA patients.

## Introduction

Obstructive sleep apnea (OSA) is a common disease with an estimated prevalence of 3 to 7% worldwide, and is the most common form of sleep disordered breathing (Franklin and Lindberg, 2015). OSA is characterized by periodic and repetitive partial or complete collapse of the upper airway during sleep, resulting in intermittent hypoxia, hypercapnia, sleep fragmentation, increased sympathetic activity and altered immunity (Strollo and Rogers, 1996; de Lima et al., 2016).

Periodontitis is a chronic infectious disease caused by pathogenic bacteria that can initiate the inflammatory response of the host (Marchesan et al., 2020). The synergetic effect of both bacterial toxins and host immune response results in the destruction of the supporting tissues of the teeth, clinically manifested as progressive loss of periodontal attachment, pocket formation, loss of alveolar bone and, ultimately, tooth loss (Cekici et al., 2014).

Recent studies have revealed an increased risk of periodontitis in patients with OSA compared to healthy controls (Al-Jewair et al., 2020). As one explanation of the association between OSA and periodontitis states, the inflammatory response of OSA could enhance the inflammatory status of current diseases or trigger inflammatory diseases in the host (Ryan et al., 2005). In periodontitis, a chronic inflammation of the periodontal tissue is initiated by periodontal pathogens when a biofilm accumulates proximal to the gingiva (Mira et al., 2017). Substances such as lipopolysaccharides and toxins, which are produced by the microbiome in the biofilm, can activate the immune response of the host, leading to the release of various cytokines and inflammatory mediators (Darveau, 2010; Yucel-Lindberg and Båge, 2013). Consequently, we hypothesized that OSA could initiate the pathogenesis of periodontitis by altering the microbial community surrounding the periodontium.

To analyze the microbial composition of biofilms, previous studies reported that using saliva samples is more suitable, as saliva is more easily and noninvasively obtained than dental plaque samples (Lundmark et al., 2019). Moreover, the periodontal pathogens present in subgingival plaque are reflected by the salivary microbial composition (Yoshizawa et al., 2013; Belstrøm et al., 2018). A significant positive correlation was detected between saliva samples and subgingival plaque samples in patients with periodontitis (Haririan et al., 2014), emphasizing the value of saliva as a diagnostic fluid.

16S ribosomal RNA (rRNA) gene sequencing is used to detect the abundance and diversity of microorganisms and to identify species efficiently and precisely in many environments, including salivary samples (Johnson et al., 2019). Accordingly, in this study, we used high-throughput 16S rRNA gene sequencing to characterize the microbial profiles of saliva samples of patients with OSA compared with non-OSA controls to determine whether the microbial composition of biofilms in subjects with OSA was altered. We also investigated the distribution of specific periodontal pathogens in the two groups to seek an explanation for the increased risk of periodontitis in OSA.

## Materials and Methods

### Participants

This study was approved by the Ethics Committee of the Airforce Medical Center and the Ethics Committee of Peking University School and Hospital of Stomatology (No. PKUSSIRB-202164061). To estimate the sample size, we used the RnaSeqSampleSize method implemented in the R language by Zhao et al. which was based on the distributions of average gene read counts and dispersions estimated from real RNA-seq datasets of The Cancer Genome Atlas (TCGA) (Zhao et al., 2018). The estimated minimum fold change in prognostic gene expression between the two groups was set to 2.5 based on a former study investigating the salivary microbiome of OSA patients and normal controls (Jia et al., 2020), and the sample size of 50 would reach a false discovery rate (FDR) of 0.01 with a total of 20,000 genes for testing and 200 prognostic genes. Considering possible complications of the study, 60 participants were initially recruited.

From July to December 2019, patients aged 25–35 years who were diagnosed with obstructive sleep apnea were included from two sleep medicine centers in Beijing. Then, non-OSA controls matched with the OSA group by age, gender and BMI were voluntarily recruited from an enterprise’s male dormitory in Beijing. The inclusion criteria were as follows: 1) age >18 years; 2) male; 3) self-reported as having no alcohol abuse; and 4) self-reported as never smoking or having quitted smoking for more than 6 months. All participants signed informed consent after being told study details.

The exclusion criteria included a special diet preference, pet feeding, systemic diseases (hypertension, diabetes, etc.), salivary gland disease, oral mucosa infectious disease, untreated decays or pericoronitis, application of antibiotics or hormone drugs in the last 3 months, and periodontal and/or OSA treatment in the last 1 month. Among the 60 initially recruited participants, two voluntarily withdrew the periodontal examination, one failed to record sleep monitoring data due to connection failure, and three had saliva samples containing less than 0.5 ng/μl DNA. Finally, 54 participants, consisting of 27 OSA patients and 27 normal controls, completed the whole study with a dropout rate of 10%.

### Periodontal Examination

The periodontal examination was practiced for each participant by one experienced double-blinded dentist before polysomnography (PSG). A full mouth probing was conducted to record the probing depth (PD) and clinical attachment level (CAL) of each participant. All measurements were obtained using a Williams style periodontal probe (Hu-Friedy, Inc., Chicago, IL, USA) and lengths were rounded up or down to the nearest millimeter. CAL is determined as follows: 1) If the gingival margin is located on the anatomic crown, CAL is calculated by PD minus the distance from the gingival margin to the cementoenamel junction (CEJ). 2) If gingival margin coincides with the CEJ, CAL equals to PD. 3) If the gingival margin is located apical to the CEJ, CAL is PD plus the distance between the CEJ and the gingival margin.

In the diagnosis of periodontitis, we used the 2017 World Workshop on the Classification of Periodontal and Peri-implant Diseases and Conditions. A patient was a periodontitis case if: 1) Interdental CAL is detectable at ≥2 non-adjacent teeth, or; 2) Buccal or oral CAL ≥3 mm with pocketing >3 mm is detectable at ≥2 teeth (Tonetti et al., 2018).

### Diagnosis of OSA

Each participant underwent standard overnight PSG using a portable computerized device (SOMNOscreen™ plus, SOMNOmedics GmbH, Randersacker, Germany).

Before PSG, the blood pressure and heart rate were recorded. Accordingly, central electroencephalogram and electrocardiogram were taken. Chest and abdominal movement were traced by respiratory effort bands. Body position and oronasal airflow were recorded by pressure sensors, and oxygen saturation (SaO_2_) was measured by a pulse oximeter. In the morning, blood pressure and heart rate were recorded again within 1 h after the rising.

Sleep stages and arousals, movements, and cardiopulmonary events were manually scored by one experienced sleep technician based on the scoring manual of the American Academy of Sleep Medicine (AASM) (Berry et al., 2020). The standard definition of an apneic event includes a minimum 10-second interval between breaths, with a neurologic arousal, a blood oxygen desaturation of 3% or greater, or both arousal and desaturation. Hypopnea is defined as an episode of shallow breathing (airflow reduced by ≥50%) during sleep, lasting for ≥10 s and usually associated with a fall in blood oxygen saturation attributable to partial obstruction of the upper airway. The apnea–hypopnea index (AHI) is calculated as the total number of apneas and hypopneas per hour of sleep. In adults, the presence of OSA was determined as an AHI score of ≥5, while mild OSA is defined as an AHI of at least 5 to 15 events per hour, moderate OSA as >15 to 30 events per hour, and severe OSA as >30 events per hour (Sateia, 2014).

### Saliva Collection

Saliva samples were collected in the morning after the overnight PSG. All participants were required not to drink, eat, brush teeth, or floss for at least 2 h. Fifteen minutes before sampling, they were guided to gently rinse their mouth using purified water. Approximately 2 ml of whole saliva was collected by passive drool using Saliva Collection Aid (Salimetrics, State College, PA, USA). All samples were frozen at −20°C immediately after collection, and transferred to −80°C refrigerators in no more than 4 h until further processing.

### DNA Extraction and Sequencing

Before DNA extraction, all samples were thawed at room temperature. Approximately 500 μl saliva of each sample was transferred into the centrifuge tube and centrifuged at 10,000 rpm for 15 min. Only the residues were collected and resuspended in 300 μl phosphate buffered saline (PBS). Then we used QIAamp DNA Mini Kit (Qiagen, Valencia, CA, USA) to extract DNAs according to the manufacturer’s protocols. The quality of the extracted DNA was checked by the spectrophotometer (Nanodrop 8000, Thermo Fisher Scientific, Waltham, MA, USA) to see whether the OD_260/280_ ratio was 1.8–2.0. The integrity of DNA was verified by 1% agarose gel electrophoresis, where samples with less than 0.5 ng/μl DNA were excluded. The high-quality DNA was stored at −20°C for further sequencing.

Isolated DNA was used as a template for polymerase chain reaction (PCR) (Ko et al., 2019). The V3–V4 regions of the bacterial 16S rRNA gene were amplified with 341’F primer (CCTAHGGGRBGCAGCAG) and 805’R primer (GACTACHVGGGTATCTAATCC) (Hugerth et al., 2014) using a KAPA HotStart ReadyMix PCR Kit (KAPA Biosystems, Wilmington, MA, USA). The PCR program was set as follows: 98°C for 2 min, 26 cycles of 98°C for 20 s, 54°C for 20 s, 72°C for 2 min (Eriksson et al., 2019). The products were purified using an AxyPrep DNA Gel Extraction Kit (Axygen, Union City, CA, USA). The libraries were sequenced using the Illumina NovaSeq™ 6000 platform (Illumina Inc, San Diego, CA, USA) by Shanghai Personalbio Co., Ltd. (Shanghai, China) and all sequencing data were submitted to the NCBI Short Reads Archive Database under accession number SRP339526.

### 16S rRNA Gene Library Preparation and Data Processing

To process the raw sequencing data, the Vsearch (v2.13.4_linux_x86_64) software was used. First, amplicon reads with low quality and primers were trimmed and filtered, followed by chimeras removed and singletons filtered. Then selected high-quality sequences with a similarity threshold of 97% were clustered to the same operational taxonomic units (OTUs) using UPARSE (Edgar, 2013). In each OTU, the most abundant sequence was used as the representative sequence and was taxonomically classified into microbial taxa (phylum, class, order, family, and genus) according to the Ribosomal Database Project database and the Human Oral Microbiome Database (Dewhirst et al., 2010).

To compare the diversity or complexity of species in OTUs from OSA and non-OSA samples, alpha diversity analysis was applied through several indices, namely, observed species, Chao, Shannon, Simpson, Pielou’s evenness and goods coverage. The indices were calculated by Mothur (v1.31.2), and one-way ANOVA analysis was used for multigroup comparison. In beta diversity analysis, we used the unweighted UniFrac distances matrices to perform the principal coordinates analysis (PCoA) and analysis of similarities (ANOSIM), in order to ordinate the dissimilarity matrices of the samples.

To analyze the difference of species components in each group, the number of total OTUs, OTUs in the OSA and the non-OSA group was shown by a Venn diagram. Then, the distribution of OTUs in each sample were plotted in one histogram using R, version 3.0.3 (R Foundation for Statistical Computing, Vienna, Austria). Kruskal–Wallis tests were conducted to determine which taxonomic groups were significantly different between groups of samples.

Multivariate analyses using Multivariate Association with Linear Models (MaAsLin) was performed to test associations between OSA and salivary microbiome, taking periodontitis, age, BMI, and alcohol use into account as possible confounding factors. In the MaAsLin models, periodontitis (yes/no) and alcohol use (current/former/never) were defined as categorical metadata variables, along with age and BMI defined as continuous variables. A q-value <0.10 was used as significance cut-off for this taxa-phenotype associations (van der Meulen et al., 2019).

### Statistical Analysis

All statistical analysis, if not specified, was conducted using IBM SPSS Statistics for MacOS, version 26.0 (IBM Corp., Armonk, N.Y., USA), and a two-sided p-value of <0.05 was considered to be statistically significant. A normality test was practiced on all variances we collected.

We set OSA as the risk factor and periodontitis as the outcome, and the χ² test was applied to measure the difference in prevalence of periodontitis between variables. The differences between the mean values of PD were then evaluated between two groups using the independent t-test.

The comparison of sequencing data between two groups were firstly tested for the homogeneity of variance, and then followed by one-way ANOVA or Wilcoxon rank sum test.

## Results

### Characteristics of the Study Participants

In total, 54 male participants were enrolled in the study, with 27 participants in the OSA group and 27 participants in the non-OSA group according to the PSG diagnosis. The two groups were comparable with respect to other characteristics, as age, alcohol use, annual incomes, and level of education were basically matched. Additional participants demographics are presented in **Table 1**.

**Table 1:** Demographic characteristics of the study samples grouped by OSA.

Demographic characteristics	All Participants (n = 54)	OSA Yes (n = 27)	OSA No (n = 27)	p-Value
Age (years)	28.5 ± 3.1	27.9 ± 3.2	29.3 ± 2.8	0.120
24–29	33 (61.1)	15 (55.6)	18 (66.7)	
30–35	21 (38.9)	12 (44.4)	9 (33.3)	
BMI (kg/m^2^)	24.4 ± 2.6	25.6 ± 2.3	23.2 ± 2.4	<0.001^*^
<18.5	1 (1.9)	0 (0)	1 (3.7)	
18.5–24.9	28 (51.9)	10 (37.0)	18 (66.7)	
25.0–29.9	25 (46.3)	17 (63.0)	8 (29.6)	
Alcohol Use				
Never	18 (33.3)	8 (29.6)	10 (37.0)	
Former	2 (3.7)	2 (7.4)	0 (0)	
Current	34 (63.0)	17 (63.0)	17 (63.0)	
AHI (times/h)	5.08 ± 3.20	2.38 ± 1.39	7.79 ± 1.94	<0.001^*^
Periodontitis				0.028^*^
Yes	30 (55.6)	19 (70.4)	11 (40.7)	
No	24 (44.4)	8 (29.6)	16 (59.3)	
Periodontal Parameters				
mean PD (mm)	3.09 ± 0.41	3.13 ± 0.44	3.04 ± 0.38	0.489
CAL (mm)	2.31 ± 0.60	2.35 ± 0.67	2.27 ± 0.54	0.625

Values are shown as mean ± SD or n (%).

^*^p < 0.05 was considered statistically significant.

With regard to the PSG diagnosis, almost all participants could be considered normal range (51.9% had BMI from 18.5 to 24.9 kg/m^2^) or pre-obese (46.3% had BMI from 25 to 29.9 kg/m^2^) according to the WHO criteria for obesity. A significantly higher BMI and a higher proportion of pre-obese participants were discovered in the OSA group.

Based on the periodontal examination, 30 participants (55.6%) had been diagnosed with periodontitis. The OSA group showed a significantly higher prevalence of periodontitis, but there was no statistically significant difference of mean PD or mean CAL between the two groups.

### 16S rRNA Gene Amplicon Sequencing Data

**Figure 1:**
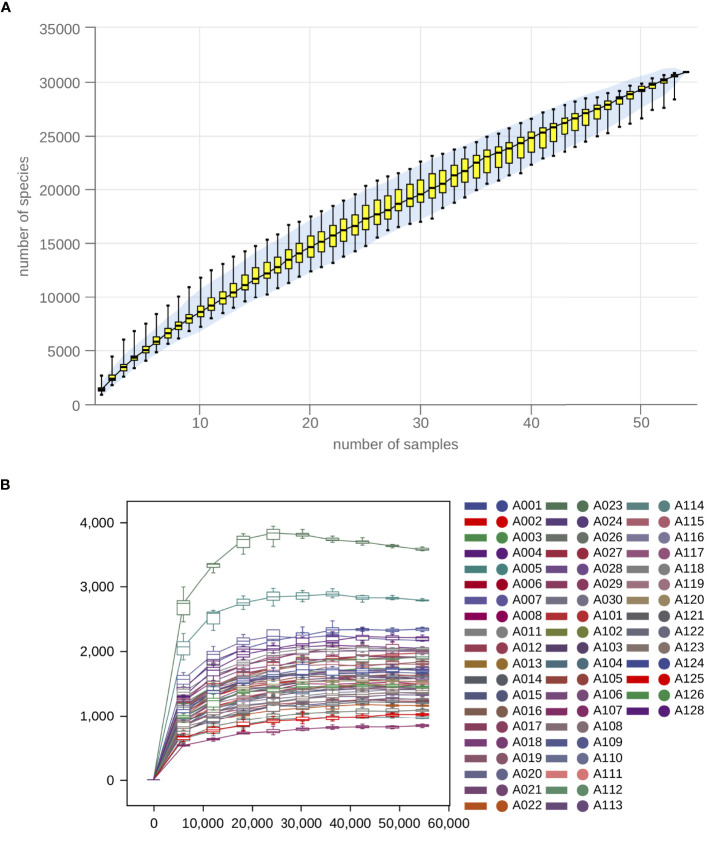
Species accumulation and rarefaction analysis indicated that the sample size is sufficient enough to represent the species composition. **(A)** Sample-based species accumulation curve and **(B)** Sample-based rarefaction curve gradually became flat with the increasing number of sequencing samples.

After data processing, a total of 4,133,794 high-quality reads were generated from 54 salivary samples (ranging from 57,599 to 112,104 reads), with an average of 76,552 ± 10,625 reads per sample. Then, all reads were clustered by using a 97% similarity cutoff. A total of 483 OTUs, with 341 and 434 OTUs in the OSA and the non-OSA group respectively were finally detected. As shown by the species accumulation curve (**Figure 1A**) and the rarefaction curve (**Figure 1B**), the curve gradually became flat with the increasing number of sequencing samples, indicating that the sample size is sufficient enough to represent the species composition as well as the diversity of microorganisms in the samples.

### Alpha Diversity Analysis

**Figure 2:**
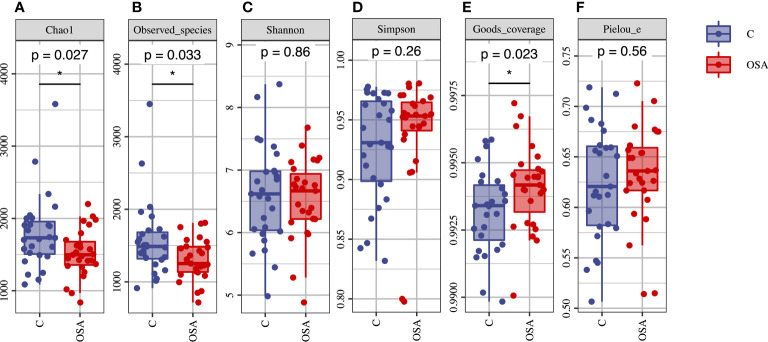
Alpha diversity analysis of the salivary microbial samples. **(A)** Chao1 Index; **(B)** Observed species; **(C)** Shannon Index; **(D)** Simpson Index; **(E)** Goods coverage; **(F)** Pielou’s evenness. The Chao1 and observed species were significantly decreased in the OSA group. Goods coverage were significantly higher in the OSA group. There was no significant difference of Shannon, Simpson and Pielou’s evenness between the two groups. *p < 0.05 was considered statistically significant.

The alpha diversity of microbial community was calculated and then compared between the OSA group and the non-OSA group. The OSA group had a significantly lower microbial species richness than the controls, as demonstrated by the Chao index (p = 0.027) (**Figure 2A**) and observed species (p = 0.033) (**Figure 2B**). The OSA group also showed higher goods coverage (p = 0.023) (**Figure 2E**). Shannon (**Figure 2C**) and Simpson (**Figure 2D**) indexes indicated that there was no statistical difference of the microbial species diversity between the two groups. The microbial species evenness estimator (Pielou’s evenness) (**Figure 2F**) also showed no significant difference.

### Beta Diversity Analysis

**Figure 3:**
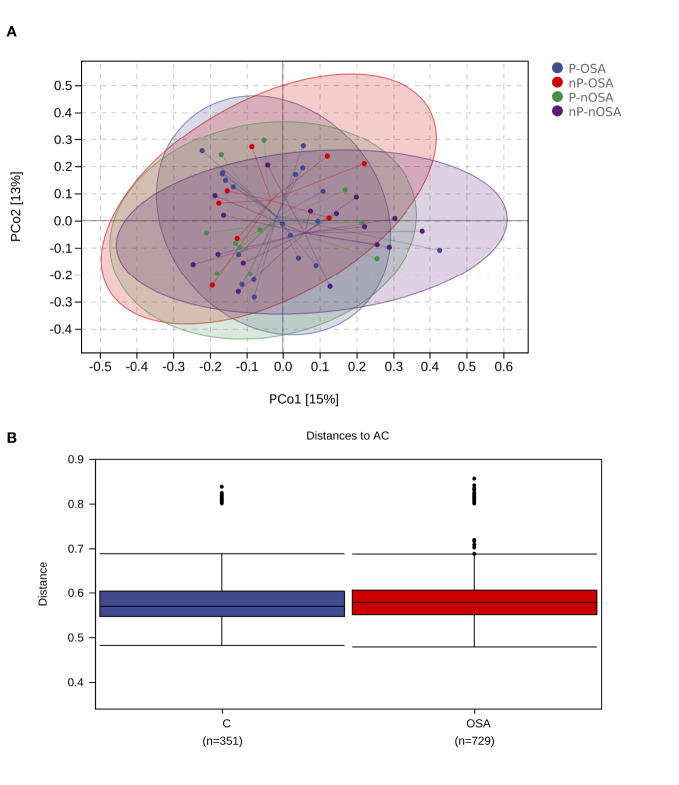
Beta diversity analysis of the salivary microbial samples. **(A)** Principal Coordinates Analysis (PCoA) calculated by the relative abundance of OTUs. Salivary microbial samples were divided into four categories. OSA-P, OSA/periodontitis; OSA-nP, OSA/non-periodontitis; nOSA-P, non-OSA/periodontitis; nOSA-nP, non-OSA/non-periodontitis. The communities of the four categories had the tendency to cluster apart from each other. **(B)** The average weighted UniFrac distance value of the OSA group is slightly higher than the non-OSA group.

Beta diversity analysis was conducted to characterize the similarities or differences of microbial communities. PCoA to unweighted UniFrac distances were conducted in four categories: OSA/periodontitis, OSA/non-periodontitis, non-OSA/periodontitis, and non-OSA/non-periodontitis. A separation was observed among the four categories. Scatters of the OSA/non-periodontitis gathered to the left top of the plot, while scatters of the non-OSA/non-periodontitis category gathered to the right bottom (**Figure 3A**).

The distinction of microbial communities between the two groups were further tested by ANOSIM analysis (**Figure 3B**), showing a significant higher between-habitat diversity at the OSA group than that at the non-OSA group (R^2^ = 0.065875, p = 0.005).

### Microbial Distribution

**Figure 4:**
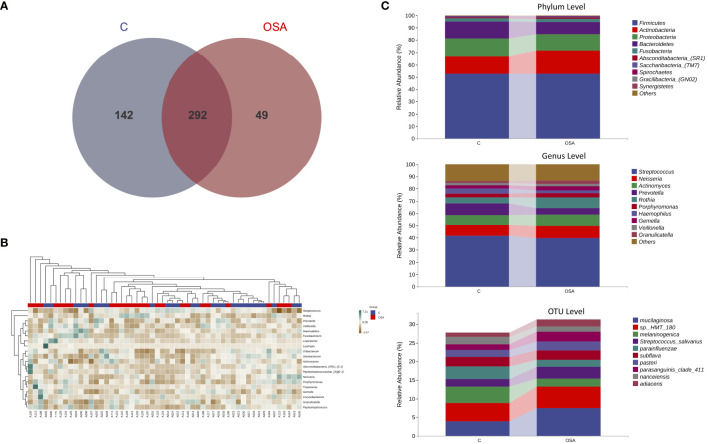
Analysis of the composition and structure of microbial communities at the genus level. **(A)** Venn diagram to visualize the shared and unique OTUs in the OSA and the non-OSA group. **(B)** Heatmap of the relative abundance of salivary microbiome at the genus level of each participant. **(C)** The relative abundance and distribution displaying the top-most significantly different taxa at phylum, genus, and OTU levels between the OSA and the non-OSA group.

As shown by the Venn diagram (**Figure 4A**), the number of total OTUs in the OSA group was fewer than the non-OSA group (341 versus 434). There were 49 unique OTUs in the OSA group and 142 in the non-OSA group.

The taxonomic composition in samples of genus level was then generated into the heatmap (**Figure 4B**) to visualize the distribution of bacteria in each sample. While analyzing the detected bacteria at phylum, genus, and OTU levels, the microbial distribution of the top 10 taxa is provided in **Figure 4C**. The main detected bacteria phylum in the two groups included *Firmicutes*, *Actinobacteria*, and *Proteobacteria*, which made up more than 70% of the whole bacteria in each group. The top-most genus included *Streptococcus*, *Neisseria*, and *Actinomyces*. It was also observed that the microbial community structure differed from the OSA group to the non-OSA group. Among these top abundant microbes, *Rothia* (8.73% versus 5.01%) and *Gemella* (3.58% versus 2.56%) showed a significant abundance in the OSA group. In contrast, the abundance of *Streptococcus* (4.07% versus 2.17%) and *Veillonella* (4.07% versus 2.17%) was relatively higher in the non-OSA group. In OTU level, *R. mucilaginosa* (7.39% versus 3.92%) was significantly abundant in the OSA group, while the abundance of *P. melaninogenica* (4.36% versus 2.12%) was higher in the non-OSA group.

In the MaAsLin models, OSA was statistically associated with *Peptostreptococcus* and *Granulicatella* (q <0.1). No statistical associations were detected between OSA and periodontitis, age, BMI, and alcohol use.

### Periodontal Pathogens

**Figure 5:**
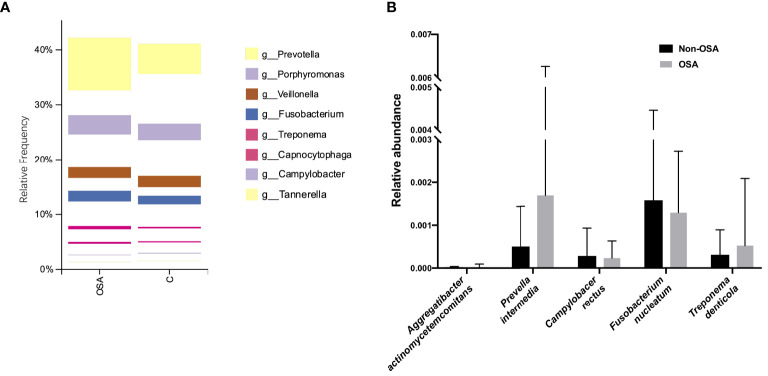
Analysis of the relative abundance of specific genus and species related to periodontitis. **(A)** The relative abundance of the periodontal pathogens at the genus level. Only *Prevotella* was more abundant in the OSA group. **(B)** The relative abundance of the periodontal pathogens at the species level. No significant difference was observed in the selected five species between two groups.

At the genus level, the relative abundance of eight predominant genera which had been proved to initiate periodontal disease was visualized in the plot (**Figure 5A**). Further statistical analysis showed that only *Prevotella* were observed to have significant higher abundance in the OSA group than that in the non-OSA group. The relative frequency of other seven genera were found to have no significant difference between the OSA group and the non-OSA group (**Table 2**).

**Table 2:** Relative frequencies of eight genus in the OSA and the non-OSA groups.

Genus	OSA group	Non-OSA group	p-Value
*Prevotella*	0.0570 ± 0.0446	0.0970 ± 0.0722	0.018^*^
*Porphyromonas*	0.0334 ± 0.0253	0.0301 ± 0.0235	0.625
*Veillonella*	0.0199 ± 0.0142	0.0215 ± 0.0175	0.709
*Fusobacterium*	0.0178 ± 0.0199	0.0165 ± 0.0246	0.827
*Treponema*	0.0051 ± 0.0146	0.0026 ± 0.0042	0.400
*Capnocytophaga*	0.0032 ± 0.0034	0.0022 ± 0.0017	0.185
*Campylobacter*	0.0009 ± 0.0008	0.0015 ± 0.0021	0.172
*Tannerella*	0.0005 ± 0.0009	0.0006 ± 0.0006	0.989

Values are given as mean ± SD.

^*^One-way ANOVA p <0.05 was considered statistically significant.

At the species level, we included five specific species which have a clear proof of periodontal pathogenesis in this study, namely, *Aggregatibacter actinomycetemcomitans* (*Aa*), *Prevella intermedia* (*Pi*), *Campylobacer rectus* (*Cr*), *Fusobacterium nucleatum* (*Fn*) and *Treponema denticola* (*Td*). As shown by the box diagram (**Figure 5B**), the relative abundance of *Aa*, *Pi*, and *Td* was slightly higher in the OSA group. However, the differences of relative abundances of these five species related to periodontal diseases were not significant between the two groups (**Table 3**).

**Table 3:** Relative frequencies of five species in the OSA and the non-OSA groups.

Genus	OSA group	Non-OSA group	p-Value
*Aggregatibacter actinomycetemcomitans* (*Aa*)	0.000023 ± 0.000070	0.000007 ± 0.000032	0.297
*Prevella intermedia* (*Pi*)	0.001689 ± 0.00456	0.000501 ± 0.000935	0.191
*Campylobacer rectus* (*Cr*)	0.000233 ± 0.000396	0.000281 ± 0.000646	0.742
*Fusobacterium nucleatum* (*Fn*)	0.001288 ± 0.001441	0.001574 ± 0.002886	0.647
*Treponema denticola* (*Td*)	0.000520 ± 0.001562	0.000308 ± 0.000581	0.512

Values are given as mean ± SD.

## Discussion

The findings of this study were not completely in accordance with previous studies which indicated a stable and unchanged alpha diversity of saliva microbiome between the OSA and the non-OSA groups (Jia et al., 2020). Rather, we detected a significantly decreased microbial richness and also a higher between-habitat diversity in the OSA group, as demonstrated by alpha and beta analyses. Additionally, the total and unique OTUs also increased from the OSA to the non-OSA group. An interesting finding of the microbial community structure was that *Rothia* and *Gemella*, both gram-positive, were more abundant in the OSA group, while *Prevotella* and *Haemophilus*, both gram-negative, were more abundant in the non-OSA group. These results indicated that the structure of the saliva microbial community in the OSA group was altered in terms of species richness and trans-habitat diversity. Regarding the different species between two groups detected by the MaAsLin analysis, *Peptostreptococcus* is a recognized pathogen in medical infections, and also a potential pathogen in adult periodontitis (Rams et al., 1992). *Granulicatella* is a normal component of the oral flora, but has been associated with a variety of invasive infections in man (Cargill et al., 2012). The results implicated that the observed differences of the two species might be related to the biological variation itself, rather than periodontal conditions, age, BMI or alcohol consumption differences, and such differences might be associated with the pathogenesis of periodontitis.

The mechanism might be related to repeated episodes of collapse of the upper airway during sleep which result in intermittent hypoxia in patients with OSA (Moreno-Indias et al., 2016). Recent studies showed that hypoxia plays an important role in host’s both adaptive and innate immunity, by regulating transcription factors including nuclear factor-κB (NF-κB) and hypoxia-inducible factor (HIF) (Bhandari and Nizet, 2014). Consequently, the intermittent hypoxia caused by OSA could regulate these important immune transcription factors in the host, which significantly alters the process of subsequent disease progression and bacterial infection (Schaffer and Taylor, 2015).

A large number of *in vivo* studies support the possible protective effect of HIF-activating agents in tissue infected with gram-negative bacterial pathogens and some gram-positive strains (Elks et al., 2013; Oehlers et al., 2015). In detail, invasive pathogens are detected by toll-like receptors (TLRs) in immune cells, leading to the activation of NF-κB pathway that could promote the inflammation status in the host. Under the condition of hypoxia, the activity of HIF-1 is enhanced. The activated HIF-1 pathway could upregulate several factors that promote host’s immunity and the resolution of infection (Thompson et al., 2017). We hypothesized that there may be a protective mechanism in patients with OSA. Under intermittent hypoxia, the protective effect could make the harmful pathogens decrease, thus makes the diversity of microbial community slightly increased. However, further experiments of the molecular mechanism should be done to testify and strengthen the hypothesis as discussed.

When referred to the specific bacteria that could reflect the periodontal status, only *Prevotella* showed a significant increase in the genus level in the OSA group. *Prevotella* is Gram-negative bacteria that had recently been shown to be more abundant in patients with periodontitis than normal controls (López, 2000; Doungudomdacha et al., 2001). In the process of dental plaque biofilm accumulation, *Prevotella* perform as secondary colonizers which do not colonize tooth surfaces initially, but rather adhere to other colonizers that are already in the plaque (Szafrański et al., 2015). The maturation of the plaque includes the transition from early supragingival plaque to subgingival plaque. This process also involves a shift in the microbial population from primarily gram-positive bacteria to gram-negative bacteria. Therefore, during the later stages of plaque formation, gram-negative species tend to predominate in the plaque (Newman et al., 2018).

Additionally, the species of five well-known periodontal pathogens, namely, *Aa*, *Pi*, *Cr*, *Fn*, and *Td* were further investigated. None of them were detected to change in abundance significantly. These results indicated that OSA might cause periodontitis, not by directly increasing the relative abundance of periodontal pathogens, but by changing the microbial community structure and also affecting the interactions of different bacteria so as to alter the pathogenesis process of disease.

In this study, the OSA group and the non-OSA group were matched by gender, age, BMI, socioeconomic conditions and education experience. Besides, we set relatively strict inclusion criteria to exclude possible confounders: smoking status, drug use, poorly controlled systematic diseases, or any other interfering conditions, thus including a study sample with the least number of patient-related confounders. Therefore, quite homogeneous samples were finally included in the study, which was ideal for investigating possible influencing factors.

This preliminary study had several important limitations. First, the sample size was relatively small in this study, which may affect the study power to distinguish periodontal pathogens as differing between groups in the analyses. Further studies may include more samples to testify and broaden the findings of the current one. Second, we only included Chinese male adults of middle age in this study. The severity of both OSA and periodontitis, if any, was far milder than that in the general population. Thus, the selection of participants did not add power of the study for population generalization and the results might not reflect the genuine structure and distribution of salivary microbiome in populations of different races, genders and ages. Consequently, adequate consideration should be given when extending the results to the general population. Future studies might conduct a perspective design including interventions, or take insight into the signaling pathways in order to improve the credibility and validity of the results.

## Conclusions

In conclusion, a significant association between OSA and periodontitis was observed in this study. Moreover, the sequencing data demonstrated that the salivary microbial community was altered in the OSA group in the aspects of species richness and trans-habitat diversity, along with *Prevotella*, a specific periodontal pathogen, showing an increasing trend in patients with OSA. These findings may shed new light on the explanation for the pathogenesis of periodontitis.

## Data Availability Statement

The datasets presented in this study can be found in online repositories. The names of the repository/repositories and accession number(s) can be found below: NCBI SRA; PRJNA767516.

## Ethics Statement

The studies involving human participants were reviewed and approved by the Peking University School of Stomatology Institutional Review Board. The patients/participants provided their written informed consent to participate in this study.

## Author Contributions

YC, HG and XG conceived and designed the study strategy. YC conducted the periodontal examinations. YC, XC and XH performed saliva DNA extraction and sequencing data analysis. YD practiced medical checks, PSG, and data collection. YC wrote the manuscript and prepared the tables and figures. HG and XG reviewed and edited the manuscript. HG and XG were responsible for study supervision. All authors contributed to the article and approved the submitted version.

## Funding

This study was funded by the National Program for Multidisciplinary Cooperative Treatment on Major Diseases (Program Number: PKUSSNMP-201902).

## Conflict of Interest

The authors declare that the research was conducted in the absence of any commercial or financial relationships that could be construed as a potential conflict of interest.

## Publisher’s Note

All claims expressed in this article are solely those of the authors and do not necessarily represent those of their affiliated organizations, or those of the publisher, the editors and the reviewers. Any product that may be evaluated in this article, or claim that may be made by its manufacturer, is not guaranteed or endorsed by the publisher.
